# Homogeneity and High Concordance of *ALK* Translocation in Primary Lung Adenocarcinoma and Paired Lymph Node Metastasis

**DOI:** 10.1038/s41598-017-11453-0

**Published:** 2017-09-08

**Authors:** Wei Ma, Lei Guo, Ling Shan, Xiuyun Liu, Ning Lyu, Jianming Ying

**Affiliations:** Department of Pathology, National Cancer Center/Cancer Hospital, Chinese Academy of Medical Sciences and Peking Union Medical College, Beijing, 100021 China

## Abstract

Translocation of *anaplastic lymphoma kinase* (*ALK*) gene is an important determinator for the response to *ALK* tyrosine kinase inhibitor (TKI) in non-small-cell lung cancer (NSCLC) patients. The existence of genetic heterogeneity will affect the results of molecular testing, especially in biopsy samples from primary or metastatic sites of patients with advanced stage NSCLC. We intended to explore the heterogeneity of *ALK* gene translocation in excision specimens and to examine the existence of discordance of *ALK* status between primary tumours and corresponding lymph node metastases. A total of 106 *ALK* positive lung adenocarcinoma cases were collected for assessment of intratumour heterogeneity of *ALK* gene translocation, which were stained by the fully automated Ventana *ALK* D5F3 immunohistochemistry (IHC) analysis. In addition, the *ALK* gene translocations were evaluated in a series of 53 primary tumours and their paired lymph node metastases using *ALK* D5F3 IHC staining. The concordance rate between primary tumours and paired metastatic lymph nodes was 100%. *ALK* status was homogeneous in lung adenocarcinoma samples and was generally stable during metastasis. Therefore, *ALK* gene translocation can be measured reliably in material from either primary or metastatic tumours in lung adenocarcinoma patients.

## Introduction

Lung cancer causes the largest number of cancer related deaths worldwide. Currently, more than 85% of lung cancer cases are classified as non-small-cell-lung cancer (NSCLC). Adenocarcinoma is the most common histological type of NSCLC^1^. Although conventional chemotherapy is the main treatment for the majority of advanced NSCLC patients, new targeted therapies have been developed for a subset of patients harboring key oncogenic alterations^2^. For example, drugs targeting *epidermal growth factor receptor* (*EGFR*) mutations and *anaplastic lymphoma kinase* (*ALK*) translocations have been successfully used in clinical medicine^2, 3^. *ALK* gene translocation was first discovered in NSCLC in 2007^4^. This translocation results from a small inversion in chromosome 2p leading to aberrant *ALK* gene translocation expression in the cytoplasm and uncontrolled cellular proliferation and survival^4, 5^. The US Food and Drug Administration (FDA) have approved crizotinib and ceritinib, the first and second-generation *ALK* inhibitors respectively^6^. The identification of *ALK* gene translocation in NSCLC is the basis of targeted therapy with *ALK* inhibitors. Three methods have been utilized to detect *ALK* gene translocations: fluorescent *in situ* hybridisation (FISH), real-time reverse transcription-PCR (RT-PCR), and novel fully automated Ventana *ALK* D5F3 immunohistochemistry (IHC). Previous study by Ying *et al*. has demonstrated the 100% sensitivity and 98% specificity of the Ventana *ALK* assay, respectively^7^.

The morphology and genetics of tumour heterogeneity are topics of great interest in cancer research^8^. Recently, several solid malignant tumours have been found to be genetically heterogeneous^8–10^. For example, IHC analysis showed that *EGFR* expression in primary NSCLC tumour/metastasis had a discordance rate of 33.3%^10^. In this study, we applied Ventana *ALK* D5F3 IHC to investigate the heterogeneity of *ALK* gene translocations in excision specimens and compared the *ALK* status between primary tumours and their corresponding metastatic lymph nodes.

*ALK* gene translocation was previously found to be mutually exclusive with other driver gene mutations^11^. However, several recent reports have identified an overlap between *ALK* translocation and other driver gene mutations^12–17^. Here, we analyze the association of *ALK* gene translocation with the occurrence of other driver gene mutations by directly sequencing the *EGFR*, *KRAS*, *BRAF*, and *HER2* gene mutations and with clinicopathological characteristics.

## Results

### Characteristics of *ALK*-positive lung adenocarcinoma samples

Clinicopathological features of 106 *ALK*-immunopositive cases were compared with 90 *ALK*-immunonegative cases (Table 1). The median age of patients with *ALK*-positive was 52.5 years (28–77 years) among 51 men and 55 women. At the time of resection, 45 (42.5%), 10 (9.4%) and 51 (48.1%) patients with *ALK*-positive were in Stages I, II and III, respectively. Among these cases, 54.7% (58/106) had lymph node metastasis. *ALK*-positive cases occurred in younger patients (*p* = 0.020), and they presented at a higher clinical stage (*p* < 0.001). Compared with *ALK*-negative patients, *ALK*-positive tumours were more likely to show lymph node metastases (*p* < 0.001). There was no significant difference in smoking history and family history of cancer among these two groups of patients (*p* > 0.05).

**Table 1:** Clinicopathologic Characteristics of *ALK*-positive and *ALK*-negative Lung adenocarcinoma samples.

characteristics	ALK-positive	ALK-negative	*P*
	N = 106(%)	N = 90(%)	
**Age, years**			
Median (range)	52.5 (28–77)	61.0(41–76)	
<65	88(83.0)	62(68.9)	0.020
≥65	18(17.0)	28(31.1)	
**Sex**			0.721
Male	51(48.1)	41(45.6)	
Female	55(51.9)	49(54.4)	
**Family history of cancer**			0.689
Yes	31(29.2)	24(26.7)	
No	75(70.8)	66(73.3)	
Smoking history			0.833
Never	71(67.0)	59(65.6)	
Formal and current	35(33.0)	31(34.4)	
**Tumor status**			0.837^#^
T1	34(32.1)	27(30.0)	
T2	58(54.7)	52(57.8)	
T3	10(9.4)	6(6.7)	
T4	4(3.8)	5(5.6)	
**Lymph node status**			<0.001^&^
N0	48(45.3)	71(78.9)	
N1	9(8.5)	7(7.8)	
N2	48(45.3)	11(12.2)	
N3	1(0.9)	1(1.1)	
**Stage**			<0.001^+^
I	45(42.5)	61(67.8)	
II	10(9.4)	13(14.4)	
III	51(48.1)	15(16.7)	
IV	0(0.0)	1(1.1)	

^**#**^T1 and T2 versus T3 and T4. ^&^N0 versus N1 and 2 and 3. ^+^Stage I and II versus stage III and IV.

### Histological characteristics of *ALK*-positive lung adenocarcinoma samples

The histological characteristics of *ALK*-positive tumours are illustrated in Table 2. Although *ALK*-positive cases were not significantly associated with acinar predominant growth pattern (*p* = 0.373), they were positively associated with micropapillary (*p* < 0.001) and solid predominant growth pattern (*p* = 0.005).

**Table 2:** Histologic characteristics of *ALK*-positive lung adenocarcinoma samples.

Histologic subtype	ALK+	ALK-	*p*
	N = 106(%)	N = 90(%)	
Lepidic	5 (4.7)	7 (7.8)	0.373
Acinar	2 (1.9)	2 (2.2)	0.869
Papillary	13 (12.3)	15 (16.7)	0.380
Micropapillary	25 (23.6)	4(4.4)	<0.001
Solid	9 (8.5)	0 (0.0)	0.005
IMA	52 (49.1)	62 (68.9)	0.005

Abbreviation: IMA, invasive mucinous adenocarcinoma.

### *ALK* gene translocation status in primary tumour cells

**Figure 1:**
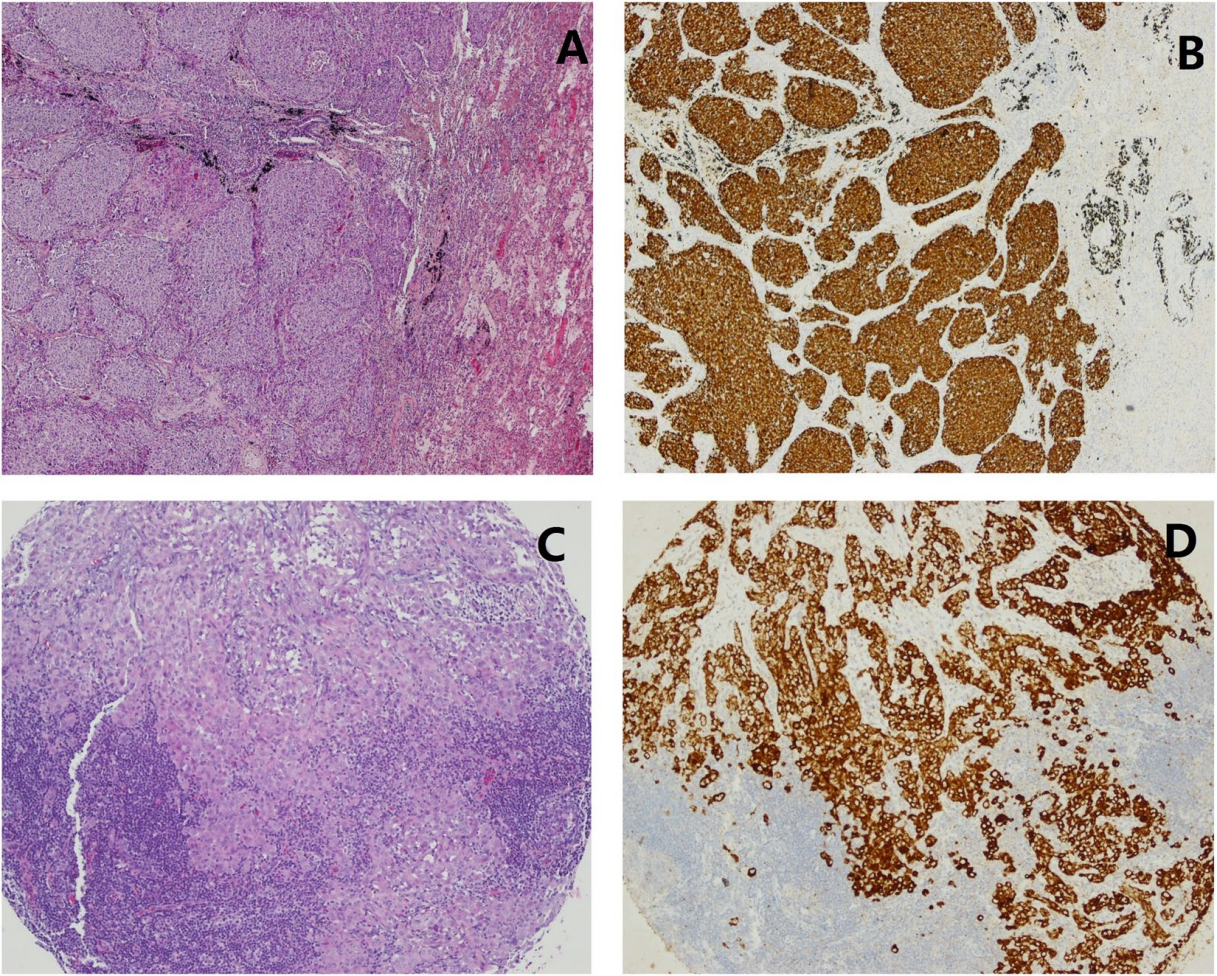
*ALK* gene status determined by the Ventana D5F3 IHC in primary tumour and corresponding lymph node metastasis. A representative case is shown: graph of the primary tumour under light microscopy (hematoxylin and eosin, ×200) (**A**) and strong immunoreactivity of *ALK*-D5F3 in the primary tumour (**B**) (×200); Graph of the corresponding lymph node metastasis under light microscopy (hematoxylin and eosin, ×200) on TMA (**C**) and strong immunoreactivity of *ALK*-D5F3 in the corresponding lymph node metastasis (**D**) (×200).

All 106 *ALK*-positive cases were successfully examined by *ALK* D5F3 IHC. All cases showed diffused cytoplasmic staining pattern in the section of FFPE tissue tumour samples, without diverse signal intensities (Fig. 1B). All *ALK*-positive cases in this study showed a homogeneous strong *ALK*-expression of all neoplastic cells.

### *ALK* gene translocation status in the lymph node metastases

Fifty three patients (53/196, 27%), including 37 *ALK*-positive cases and 16 *ALK*-negative cases, with paired primary tumours and lymph node metastases samples were further investigated for concordance of *ALK* status. Among these 53 paired sample patients, pathological N1 disease was confirmed in 10 (18.9%) patients, N2 disease in 42 (79.2%) patients and N3 disease in 1 (1.9%) patient. The characteristics of these 53 paired cases are illustrated in Table 3. Lymph node metastases from the 37 *ALK* positive cases were examined and *ALK* expression with diffused cytoplasmic staining pattern exhibited in all lymph node metastases (Fig. 1D). *ALK* expression was absent in the metastases of the 16 patients with no *ALK* expression in their primary tumours. No discordant case of *ALK* expression was observed between the primary tumours and their corresponding lymph node metastases (Table 4).

**Table 3:** Clinicopathologic characteristics of 53 paired cases.

Characteristics	N = 53	%
**Sex**		
Male	27	50.9
Female	26	49.1
**Smoking history**		
Never	34	64.1
Formal and current	19	35.9
**Family history of cancer**		
No	38	71.7
Yes	15	28.3
**Lymph node status**		
N1	10	18.7
N2	42	79.2
N3	1	2.1
**Stage**		
IIA	8	15.1
IIB	2	3.7
IIIA	40	75.5
IIIB	3	5.7

**Table 4:** *ALK* translocation in primary lung adenocarcinoma samples and their lymph node metastases by the Ventana D5F3 IHC.

Patients *ALK* translocation status in primary tumours	Patients *ALK* translocation status in metastases	
	No translocation	Translocation
No translocation	16	0
Translocation	0	37

### Analysis of *EGFR*, *KRAS*, *BRAF* and *HER2* genetic alterations in 106 *ALK*-positive cases

Genotyping for *EGFR*, *KRAS*, *BRAF* and *HER2* mutations was conducted with direct sequencing in the *ALK*-positive 106 cases. *EGFR* mutations were detected in 5 (4.7%) cases. However, *KRAS*, *BRAF* and *HER2* mutations were not detected in any specimens. *EGFR* mutation types and locations are shown in Table 5. Three cases with deletions in exon 19, whereas two had mutations in exon 21 (L858R).

**Table 5:** Clinicopathological features of patients with concomitant EGFR and ALK alterations.

Features	P1	P2	P3	P4	P5
Age	70	64	43	51	64
Sex	F	M	M	M	F
Smoking	no	yes	yes	no	no
Stage	IA	IIIA	IA	IIIA	IA
*EGFR* mutation	E19 del (2235–2249)	E19 del (2240–2257)	E19 del (2235–2257)	E21L858R	E21L858R
*ALK* Ventana IHC	+	+	+	+	+
*KRAS*	WT	WT	WT	WT	WT
*BRAF*	WT	WT	WT	WT	WT
*HER-2*	WT	WT	WT	WT	WT

Abbreviation: P, patient; F, female; M, man; Del, deletion; IHC, immunohistochemistry; WT, wild-type.

## Discussion

Tumour heterogeneity refers to the existence of subpopulations of cells with distinct genotypes and phenotypes that may harbour divergent biological behaviors, within a primary tumour and its metastases; alternatively the divergence is between tumours of the same histopathological subtype, even between spatially separated regions within single biopsies (intra- and inter- tumour, respectively)^8^. Investigation of tumour heterogeneity will provide valuable information for diagnostic and/or therapeutic procedures.

The selection of patients for crizotinib therapy relies on the *ALK* status of the tumour^18^. Thus, accurate determination of *ALK* status is crucial to ensure the potential clinical benefit of *ALK* inhibitors and to avoid the toxic effects in inappropriately selected patients^3^. The most commonly treatment-related adverse reactions in crizotinib therapy were vision disorder, gastrointestinal disorders and oedema^19^. Majority of the *ALK* gene translocation status are evaluated in primary tumours of NSCLC and in practice, most clinical decision-making for patients with advanced NSCLC depends on single-tumour biopsy samples from primary or metastatic sites, even from cytological specimens^20, 21^. Therefore, the biomarker status is assumed to remain unchanged during metastatic progression.

In the present study, we evaluated the intratumour heterogeneity of *ALK* gene translocation in 106 *ALK*-positive excision specimens by the Ventana *ALK* D5F3 IHC and also investigated whether the *ALK* status changed during disease progression in 53 pairs of primary tumours and corresponding lymph node metastases. We found the *ALK* expression was homogenous in lung adenocarcinoma samples and there was no discordant case of *ALK* status between primary tumours and corresponding lymph node metastases. Therefore, primary tumours can clearly represent the *ALK* status of the metastatic tumours. The *ALK* status remained unchanged during metastasis.

Metastatic advanced lung cancer continues to represent a major health problem worldwide. Targeted therapies are the standard therapeutic options for these patients. Approximately 70–85% of new lung cancers are unsuitable for surgical therapy; thus, only small biopsy specimens are available for diagnosis or further genetic detection^20^. By examining the *ALK* status in the primary tumours and corresponding metastatic tumours in our series, we propose that *ALK* gene translocation is a stable genetic alteration once it has occurred. This hypothesis has important clinical implications. Firstly, fine needle biopsy or aspiration of primary and/or metastatic tumours and pleural effusions are feasible for the detection of *ALK* gene translocations. Secondly, despite the marked antitumour activity of crizotinib, some *ALK*-positive patients developed resistant to crizotinib on average within the first year or two of TKI therapy^6^. In general, crizotinib resistance in *ALK*-positive NSCLC occurs through secondary resistance mutations located in the *ALK* TK domain, amplification of the *ALK* fusion gene, and activation of bypass tracks^6, 22^. Therefore, we suggested that a change in *ALK* status between the primary tumour and metastasis was not the cause of crizotinib resistance.

Previous studies have demonstrated the intratumour heterogeneity of *ALK* gene translocation in lung adenocarcinoma samples. Cai *et al*.^23^ acquired 45 spatially separated tumour cell subpopulations using laser-capture microdissection from 20 patients with *ALK*-FISH positivity. A total of 45 lesions were tested for *ALK* translocation by RT-PCR and 34 lesions were positive for *ALK* translocation, but 11 lesions were *ALK* wild-type regions. Abe *et al*.^24^ observed a total of 64 tumour areas in nine *ALK*-FISH positive cases using darkfield fluorescence microscopy; positive *ALK* was found in 50 areas. However, in the current study with the Ventana *ALK*- D5F3 IHC, we did not detect intratumour heterogeneity of *ALK* status because *ALK* protein expression was diffusely positive in all neoplastic cells. Several studies considered that the intratumoural *ALK* gene translocation heterogeneity may be associated with technical problems. Camidge *et al*.^25^ recorded the percentage of positive cells, pattern of positivity (split, single red, or both), and copy number of fused, isolated red and green signals by evaluating 90 *ALK* FISH positive NSCLC cases. They concluded that the semivertical orientation of tumour cells and/or nuclear truncation in paraffin-embedded tissue sections may make precise identification of break-apart signals difficult. Proietti *et al*.^21^ reported that FISH on conventional cytology, in which tumour cells are arranged in a single layer, have much higher positive rates for FISH than histology specimens. Moreover, *ALK* FISH using small biopsy sections cannot examine as many fields and cells as is the case with excision samples. RT-PCR, proposed as an alternative screening method for *ALK* gene translocation, is also not recommended for the detection of *ALK* translocation. RT-PCR can not be able to detect all fusion transcript variants. Additionally, RT-PCR is more stringent requirements for the sample preparation due to the rapid degradation of RNA.

The Ventana *ALK* D5F3 IHC detection system, measuring the expression of the *ALK* protein containing the C-terminal active kinase domain, is a sensitive method for detecting *ALK* gene translocation, especially in paraffin-embedded tissues, which was approved as a CE-IVD (*in vitro* diagnostic products) in Europe, China, and US since 2012, 2013 and 2015, respectively^7, 26–30^. We described a case with a negative FISH result that was later identified as *ALK*-positive by Ventana IHC and responded well to crizotinib. Targeted next generation sequencing revealed a new *ALK* partner gene (*BIRC6*) and the paracentric inversion for generating this fusion gene^31^. Wekken A. *et al*.^30^ evaluated tumour response rate and survival after crizotinib treatment of 29 consecutive patients with *ALK*-positive advanced NSCLC diagnosed by FISH and/or Ventana *ALK* D5F3 IHC on small biopsies or fine needle aspirations (FNA). They concluded that Ventana *ALK* D5F3 IHC was superior to *ALK*-FISH on small biopsies and FNA to predict tumour response and survival to crizotinib for advanced NSCLC patients. Therefore, the Ventana IHC assay may be more sensitive than FISH for detecting *ALK* status in metastases and/or small biopsies which often contain only few tumour cells.

In this study, we found that a portion of lung adenocarcinoma patients with concomitant *EGFR* and *ALK* alterations. The dual-positive cases accounted for 4.7% (5/106) of *ALK*-positive lung adenocarcinoma samples using direct sequencing, which was similar to the ratio of 4.4% (4/91) reported by Won *et al*.^13^ but lower than the ratio of 18.6% (12/70) reported by Yang *et al*.^17^; both studies used the same detection method. Won *et al*.^13^ reported that the frequency of co-alteration detection increase in the same cohort when sensitive detection methods for *EGFR* mutation are applied, such as real-time PCR, targeted NGS, and mutant-enriched NGS. We reviewed the literatures and summarized the relevant clinicopathological and molecular characteristics of *ALK/EGFR* dual-positive cases; majority of cases were adenocarcinomas in the advanced stage and mainly involved Asian patients, most *EGFR* mutations were deletions in exon 19 and point mutations in exon 21, and patients showed differential sensitivities to *EGFR*-TKI and/or *ALK*-TKI^13, 14, 16, 17^.

Intratumour heterogeneity (ITH) has recently been elucidated in several cancer types with the use of next-generation sequencing (NGS) approaches^23, 32–35^. Clonal analyses by de Bruin *et al*.^34^, and Zhang *et al*.^33^ reported substantial intratumour heterogeneity (ITH) within lung adenocarcinoma samples, by using multiregion whole-exome sequencing (WES) and/or whole genome sequencing (WGS). Recent evidence supports a model of trunk-branched clonal evolution leading to variable ITH and complex clonal architecture of tumours^33, 35, 36^. The clonal structure of a tumour is visually represented as a phylogenetic tree, the ubiquitous alterations present in all tumour regions map to the trunk, whereas heterogeneous events present in only some regions of the tumour map to the branches. The findings of homogeneous *ALK* gene translocation in lung adenocarcinoma samples, and concordant *ALK* status between primary and lymph node metastasis in this study, suggested that *ALK* gene translocation is a ubiquitous event, must occur very early in lung adenocarcinoma pathogenesis^33^. The finding of *ALK*/*EGFR* coaltered lung adenocarcinoma cases may be explained by clonal evolutionary dynamics and the resulting complex clonal architecture of lung adenocarcinoma samples. In addition, in this study, we also demonstrated that *ALK*-positive cases more commonly showed lymph node metastasis and presented at higher clinical stage, and *ALK* gene translocations were significantly more common in lung adenocarcinoma samples with micropapillary and solid predominant patterns which associated with poorer prognosis^37^. We confirmed that tumour cells with *ALK* gene translocations contained enhanced metastatic potential and acquired the metastatic phenotype.

## Conclusion

In summary, using the Ventana (D5F3) IHC, we found the homogeneity of *ALK* expression in lung adenocarcinoma samples and concordance in *ALK* status between primary tumours and corresponding lymph node metastases. Molecular testing for *ALK* translocation may be performed in either primary tumour or lymph node metastasis samples from the same patient. However, the main limitation in our study is that the metastases did not include different metastatic sites, especially distant metastases. Loco-regional or concurrent regional lymph node metastases are not considered to be biologically equivalent to distant metastases^9^. We also found a small portion of lung adenocarcinoma samples have concomitant *EGFR* and *ALK* alterations by using direct sequencing. The clinical relevance of these concurrent alterations remains to be elucidated in future studies.

## Methods

### Patients and tumour samples

All formalin-fixed and paraffin-embedded (FFPE) tissue sections were obtained from patients with histologically confirmed primary lung adenocarcinoma. The patients previously underwent curative surgery at the Cancer Hospital, Chinese Academy of Medical Sciences, Beijing, China, between February 2013 and February 2015. Written informed contents were obtained from all subjects before collecting the samples. All the methods were carried out in accordance with the institutional guidelines and approved by the Ethical Review Committee of the Cancer Hospital, Chinese Academy of Medical Sciences, Beijing, China. Tumours were immunoassayed with *ALK* D5F3 antibody as described below. A total of 106 *ALK-* immunopositive cases were collected. Ninety *ALK*-immunonegative lung adenocarcinoma cases were randomly selected and included in the study as controls.

The *ALK* status between primary tumours and their metastases was compared in 37 *ALK*-immunopositive and 16 *ALK*-immunonegative cases, as well as their corresponding metastatic lymph nodes. Thirty-seven *ALK*-positive cases with their corresponding metastatic lymph nodes were selected among 106 *ALK*-positive cases, meanwhile, 16 *ALK*-negative cases with their corresponding metastatic lymph nodes were selected among 90 *ALK*-negative cases. Our study included only metastatic tumour tissues with diameters greater than 0.5 cm from lymph nodes. The lymph node metastases of 53 cases were conducted into three tissue microarrays (TMA). Lymph node metastases were sampled by collecting 2.0 mm-diameter cores from two different representative sites based on hematoxylin and eosin (HE) stained sections.

Age, gender, cancer stage, smoking status and treatment of all patients were recorded. All cases had one slide stained for hematoxylin and eosin to confirm the presence of adequate tumour tissue. For each case, multiple slides corresponding to whole tissue sections were reviewed by two pathologists according to the 2011 International Association for the Study of Lung Cancer (IASLC)/American Thoracic Society (ATS)/European Respiratory Society (ERS) International Multidisciplinary Classification of Lung Adenocarcinoma and the American Joint Committee on Cancer (7th edition) of tumour, node and metastasis staging criteria^38, 39^. In mixed-subtype adenocarcinoma samples, we assessed the percentage of each histological pattern (Lepidic, Acinar, Papillary, Micropapillary, Solid) in 5% increments and recorded the predominant histological pattern^38^. Cases with differences between the two reviewers were reevaluated and a consensus interpretation was rendered. None of patients had received prior *ALK* or *EGFR* TKI therapy.

### Ventana IHC staining and scoring

*ALK*-IHC was performed on 4 μm-thick formalin fixed, paraffin-embedded tissue sections or TMA slides using the Ventana *ALK* D5F3 CDx assay on a Ventana Benchmark XT automated slide-processing system (Ventana Medical Systems Inc., Tucson, AZ). Briefly, slides of lung adenocarcinoma tumours were subjected to deparaffinization using EZ Prep (Ventana Medical Systems Inc.). Tissue sections were incubated with anti-*ALK* antibody (clone D5F3, Ventana Medical Systems Inc.) for 20 min. Optiview DAB IHC detection kit (Ventana Medical Systems Inc.) and Optiview amplification kit (Ventana Medical Systems Inc.) were used according to the manufacturer’s recommendations for the visualization of the bound primary antibody^27^. Tissue slides were counterstained with hematoxylin II and Bluing Reagent (Ventana Medical Systems Inc.). An *ALK*-positive cell lines embedded in agar/FFPE or sections of normal appendix containing *ALK*-positive ganglion cells, were used as *ALK*-IHC external controls in each run (Ventana Medical Systems Inc.). Two consecutive 4μm-thick FFPE tissue slides was cut, one slide was used in *ALK*-D5F3 IHC analysis, and the other for routine negative control staining for a matched rabbit monoclonal negative antibody (Ventana Medical Systems Inc.). For evaluating the staining results, a binary scoring system (positive or negative for *ALK* status) was used (package insert for VENTANA anti-*ALK* D5F3 Rabbit Monoclonal Primary Antibody, Cat.N0.790-4794/06679072001). Tumour cells with strong granular cytoplasmic staining (any percentage of positive tumour cells) were designated as *ALK*-positive, whereas tumour cells without strong granular cytoplasmic staining were designated as *ALK*-negative. Negative quality control sections were first evaluated for lack of staining^27^. Considering that Ventana *ALK* D5F3 IHC produced more intense cytoplasmic signals because of excessive chromogen deposition, which resulted in false-positive staining^40^, the *ALK* expression in our study was assessed independently by one trained scientist (J.Y) and one pathologist (N.L).

### Direct sequencing of *EGFR*, *KRAS*, *BRAF* and *HER2*

Direct Sanger sequencing for *EGFR*, *KRAS*, *BRAF* and *HER2* was conducted. Briefly, genomic DNA was extracted from 106 *ALK*-positive FFPE tissues. Mutations in exons 18–21 of *EGFR*, *V600E* of *BRAF*, exon 2 of *KRAS*, and exon 20 of *HER2* were amplified by PCR. PCR products were purified with QIA quick PCR purification kits (Qiagen, Hilden, Germany) and submitted for sequencing with an ABI 3500XL analyser (Applied Biosystems, Caarlsbad, CA, USA) with the POP7 polymer according to the manufacturer^’^s protocol. Details of the methodology were described in a previous study^41^.

### Statistical analysis

Fisher’s exact test was used to compare categorical data for clinicopathological characteristics between *ALK*-immunopositive and *ALK*-immunonegative subgroups. All *p* values are based on two-sided hypothesis test. The statistical analysis were conducted using SPSS version 17.0 software (SPSS, Chicago, IL, USA), and statistical significance was set as *p* < 0.05.

## References

[1] Torre LA (2015). Global cancer statistics, 2012. CA: a cancer journal for clinicians.

[2] Sholl LM (2015). Multi-institutional Oncogenic Driver Mutation Analysis in Lung Adenocarcinoma: The Lung Cancer Mutation Consortium Experience. Journal of thoracic oncology: official publication of the International Association for the Study of Lung Cancer.

[3] Sasaki T, Rodig SJ, Chirieac LR, Janne PA (2010). The biology and treatment of EML4-ALK non-small cell lung cancer. European journal of cancer.

[4] Soda M (2007). Identification of the transforming EML4-ALK fusion gene in non-small-cell lung cancer. Nature.

[5] Palmer RH, Vernersson E, Grabbe C, Hallberg B (2009). Anaplastic lymphoma kinase: signalling in development and disease. The Biochemical journal.

[6] Shaw AT, Engelman JA (2013). ALK in lung cancer: past, present, and future. Journal of clinical oncology: official journal of the American Society of Clinical Oncology.

[7] Ying J (2013). Diagnostic value of a novel fully automated immunochemistry assay for detection of ALK rearrangement in primary lung adenocarcinoma. Annals of oncology: official journal of the European Society for Medical Oncology/ESMO.

[8] Fisher R, Pusztai L, Swanton C (2013). Cancer heterogeneity: implications for targeted therapeutics. British journal of cancer.

[9] Gancberg D (2002). Comparison of HER-2 status between primary breast cancer and corresponding distant metastatic sites. Annals of oncology: official journal of the European Society for Medical Oncology/ESMO.

[10] Italiano A (2006). Comparison of the epidermal growth factor receptor gene and protein in primary non-small-cell-lung cancer and metastatic sites: implications for treatment with EGFR-inhibitors. Annals of oncology: official journal of the European Society for Medical Oncology/ESMO.

[11] Gainor JF (2013). ALK rearrangements are mutually exclusive with mutations in EGFR or KRAS: an analysis of 1,683 patients with non-small cell lung cancer. Clinical cancer research: an official journal of the American Association for Cancer Research.

[12] Tuononen K (2014). ALK fusion and its association with other driver gene mutations in Finnish non-small cell lung cancer patients. Genes, chromosomes & cancer.

[13] Won JK (2015). Concomitant ALK translocation and EGFR mutation in lung cancer: a comparison of direct sequencing and sensitive assays and the impact on responsiveness to tyrosine kinase inhibitor. Annals of oncology: official journal of the European Society for Medical Oncology/ESMO.

[14] Baldi L (2014). Concomitant EGFR mutation and ALK rearrangement in lung adenocarcinoma is more frequent than expected: report of a case and review of the literature with demonstration of genes alteration into the same tumor cells. Lung cancer.

[15] Lee JK (2012). Differential sensitivities to tyrosine kinase inhibitors in NSCLC harboring EGFR mutation and ALK translocation. Lung cancer.

[16] Chiari R (2014). Long-term response to gefitinib and crizotinib in lung adenocarcinoma harboring both epidermal growth factor receptor mutation and EML4-ALK fusion gene. Journal of clinical oncology: official journal of the American Society of Clinical Oncology.

[17] Yang JJ (2014). Lung cancers with concomitant EGFR mutations and ALK rearrangements: diverse responses to EGFR-TKI and crizotinib in relation to diverse receptors phosphorylation. Clinical cancer research: an official journal of the American Association for Cancer Research.

[18] Cui JJ (2011). Structure based drug design of crizotinib (PF-02341066), a potent and selective dual inhibitor of mesenchymal-epithelial transition factor (c-MET) kinase and anaplastic lymphoma kinase (ALK). Journal of medicinal chemistry.

[19] Curran MP (2012). Crizotinib: in locally advanced or metastatic non-small cell lung cancer. Drugs.

[20] Xiao, D. *et al*. Comparison of small biopsy specimens and surgical specimens for the detection of EGFR mutations and EML4-ALK in non-small-cell lung cancer. *Oncotarget*, doi:10.18632/oncotarget.10011 (2016). 10.18632/oncotarget.10011 PMC5312294 27322143

[21] Proietti A (2014). Anaplastic lymphoma kinase gene rearrangements in cytological samples of non-small cell lung cancer: comparison with histological assessment. Cancer cytopathology.

[22] Doebele RC (2012). Mechanisms of resistance to crizotinib in patients with ALK gene rearranged non-small cell lung cancer. Clinical cancer research: an official journal of the American Association for Cancer Research.

[23] Cai W (2015). Intratumoral Heterogeneity of ALK-Rearranged and ALK/EGFR Coaltered Lung Adenocarcinoma. Journal of clinical oncology: official journal of the American Society of Clinical Oncology.

[24] Abe H (2015). Heterogeneity of anaplastic lymphoma kinase gene rearrangement in non-small-cell lung carcinomas: a comparative study between small biopsy and excision samples. Journal of thoracic oncology: official publication of the International Association for the Study of Lung Cancer.

[25] Camidge DR (2012). Correlations between the percentage of tumor cells showing an anaplastic lymphoma kinase (ALK) gene rearrangement, ALK signal copy number, and response to crizotinib therapy in ALK fluorescence *in situ* hybridization-positive nonsmall cell lung cancer. Cancer.

[26] Marchetti A (2016). ALK Protein Analysis by IHC Staining after Recent Regulatory Changes: A Comparison of Two Widely Used Approaches, Revision of the Literature, and a New Testing Algorithm. Journal of thoracic oncology: official publication of the International Association for the Study of Lung Cancer.

[27] Wynes MW (2014). An international interpretation study using the ALK IHC antibody D5F3 and a sensitive detection kit demonstrates high concordance between ALK IHC and ALK FISH and between evaluators. Journal of thoracic oncology: official publication of the International Association for the Study of Lung Cancer.

[28] Minca EC (2013). ALK status testing in non-small cell lung carcinoma: correlation between ultrasensitive IHC and FISH. The Journal of molecular diagnostics: JMD.

[29] Mino-Kenudson M (2010). A novel, highly sensitive antibody allows for the routine detection of ALK-rearranged lung adenocarcinomas by standard immunohistochemistry. Clinical cancer research: an official journal of the American Association for Cancer Research.

[30] van der Wekken, A. *et al*. Dichotomous ALK-IHC is a better predictor for ALK inhibition outcome than traditional ALK-FISH in advanced non-small cell lung cancer. *Clinical cancer research: an official journal of the American Association for Cancer Research*, doi:10.1158/1078-0432.CCR-16-1631 (2017). 10.1158/1078-0432.CCR-16-1631 28183714

[31] Shan L (2015). BIRC6-ALK, a Novel Fusion Gene in ALK Break-Apart FISH-Negative Lung Adenocarcinoma, Responds to Crizotinib. Journal of thoracic oncology: official publication of the International Association for the Study of Lung Cancer.

[32] Devarakonda S, Morgensztern D, Govindan R (2015). Genomic alterations in lung adenocarcinoma. The Lancet. Oncology.

[33] Zhang J (2014). Intratumor heterogeneity in localized lung adenocarcinomas delineated by multiregion sequencing. Science.

[34] de Bruin EC (2014). Spatial and temporal diversity in genomic instability processes defines lung cancer evolution. Science.

[35] Xue R (2016). Variable Intra-Tumor Genomic Heterogeneity of Multiple Lesions in Patients With Hepatocellular Carcinoma. Gastroenterology.

[36] Anderson K (2011). Genetic variegation of clonal architecture and propagating cells in leukaemia. Nature.

[37] Tsao MS (2015). Subtype Classification of Lung Adenocarcinoma Predicts Benefit From Adjuvant Chemotherapy in Patients Undergoing Complete Resection. Journal of clinical oncology: official journal of the American Society of Clinical Oncology.

[38] Travis WD (2011). International association for the study of lung cancer/american thoracic society/european respiratory society international multidisciplinary classification of lung adenocarcinoma. Journal of thoracic oncology: official publication of the International Association for the Study of Lung Cancer.

[39] Wittekind C (2010). TNM system: on the 7th edition of TNM classification of malignant tumors. Der Pathologe.

[40] Ibrahim M (2016). ALK Immunohistochemistry in NSCLC: Discordant Staining Can Impact Patient Treatment Regimen. Journal of thoracic oncology: official publication of the International Association for the Study of Lung Cancer.

[41] Shan L (2015). Prevalence and Clinicopathological Characteristics of HER2 and BRAF Mutation in Chinese Patients with Lung Adenocarcinoma. PloS one.

